# Prevalence and antibiotics resistance of *Ureaplasma* species and *Mycoplasma hominis* in Hangzhou, China, from 2013 to 2019

**DOI:** 10.3389/fmicb.2022.982429

**Published:** 2022-09-15

**Authors:** Jingjuan Song, Xuanlan Wu, Yingying Kong, Hong Jin, Ting Yang, Xinyou Xie, Jun Zhang

**Affiliations:** ^1^Department of Clinical Laboratory, Sir Run Run Shaw Hospital, Zhejiang University School of Medicine, Hangzhou, China; ^2^Key Laboratory of Precision Medicine in Diagnosis and Monitoring Research of Zhejiang Province, Hangzhou, China

**Keywords:** *Ureaplasma* spp., *Mycoplasma hominis*, prevalence, antibiotic resistance, activity

## Abstract

*Ureaplasma* spp. and *Mycoplasma hominis*, frequent colonizers in the lower urogenital tract, have been implicated in various infections, with antibiotic resistance growing and varying regionally. This study aims to investigate the prevalence and antibiotic resistance profiles of *Ureaplasma* spp. and *M. hominis* in outpatients in Hangzhou, China, from 2013 to 2019. A total of 135,263 outpatients were examined to determine the prevalence of *Ureaplasma* spp. and *M. hominis*, including 48,638 males and 86,625 females. Furthermore, trends in antibiotic susceptibility of *Ureaplasma* spp. and *M. hominis* during 1999–2019 were analyzed. The cultivation, identification, and antibiotic susceptibility of the bacteria (ofloxacin, ciprofloxacin, erythromycin, clarithromycin, azithromycin, josamycin, tetracycline, doxycycline, and pristinamycin) were determined using the Mycoplasma IST2 kit. Our study indicated that the overall prevalence of total *Ureaplasma* spp./*M. hominis* was 38.1% from 2013 to 2019. *Ureaplasma* spp. were the most frequently isolated species (overall prevalence, 31.3%), followed by *Ureaplasma* spp./*M. hominis* coinfection (6.0%) and single *M. hominis* infection (0.8%). The prevalence of *Ureaplasma* spp. and *M. hominis* was significantly higher in females than in males, and the highest positive rates of total *Ureaplasma* spp./*M. hominis* were observed in both female and male outpatients aged 14–20 years. During 2013–2019, josamycin, tetracycline, doxycycline, and pristinamycin maintained exceptionally high activity (overall resistance rates, <5%) against both *Ureaplasma* spp. and *M. hominis*, but ofloxacin and ciprofloxacin showed limited activity (overall resistance rates, >70%). During 1999–2019, the rates of resistance to ofloxacin and ciprofloxacin increased against both *Ureaplasma* spp. and *M. hominis* but decreased to erythromycin, clarithromycin, azithromycin, tetracycline, and doxycycline against *Ureaplasma* spp. In conclusion, our study demonstrates a high prevalence of *Ureaplasma* spp. compared to *M. hominis* and *Ureaplasma* spp./*M. hominis*, and their distribution was associated with sex and age. Josamycin, doxycycline, and tetracycline are promising antibiotics that have remarkable activity against *Ureaplasma* species and *M. hominis*.

## Introduction

*Ureaplasma* species and *Mycoplasma hominis*, members of the class Mollicutes, are the smallest self-replicating and free-living organisms known, and are routinely identified as common commensal bacteria in the lower urogenital tract of healthy individuals. They are, however, sometimes implicated in various types of infections, such as chorioamnionitis, infertility, adverse pregnancy outcomes, and neonatal diseases (Waites et al., 2005; Taylor-Robinson and Lamont, 2011; Huang et al., 2015; Kletzel et al., 2018). Genital mycoplasmas can be identified in cervicovaginal and urethral specimens of 40–80% healthy humans. But they are relatively common in the urogenital tracts of sexually active adults with clinical manifestations, where *Ureaplasma* spp. and *M. hominis* can be found, with *Ureaplasma* spp. being the most prevalent (Song et al., 2014; Kasprzykowska et al., 2018; Beeton and Jones, 2019; Piscopo et al., 2020; Doroftei et al., 2021).

Both *Ureaplasma* spp. and *M. hominis* lack cell wall; thus, antibiotic therapies are restricted to those that prevent DNA replication (e.g., fluoroquinolones) and protein synthesis (e.g., macrolides and tetracyclines). The prevalence and antibiotic susceptibility profiles vary geographically, depending on antibiotic use and history of previous antibiotic exposure. Antibiotic resistance has been increasing in recent years probably due to the inappropriate use of antibiotics, which is most likely acquired through gene mutation or the acquisition of resistance determinants (Yang et al., 2020; Chalker et al., 2021). Therefore, it is critical to monitor the change of antibiotic susceptibility regularly to provide guidelines for the treatment of *Ureaplasma* spp. and *M. hominis* infections. The objective of this study was to determine the prevalence and antibiotic susceptibility of *Ureaplasma* spp. and *M. hominis* in outpatients in Hangzhou, China, from 2013 to 2019.

## Materials and methods

### Study participants

During the period of January 2013 to December 2019, a total of 135,263 outpatients were examined in the clinical laboratory at Sir Run Run Shaw Hospital, Zhejiang University School of Medicine, China. Of these, 48,638 were males aged 14–89 years, and 86,625 were females aged 14–94 years. Furthermore, 804 *Ureaplasma* spp. isolates were collected from outpatients between March and June of 1999–2004, and 1,278 isolates were obtained from outpatients with genital manifestations, such as vaginal or cervical discharge, painful or burning urination, dysuria, frequent urination, and other symptoms, between January 2005 and December 2012, to determine the trend in the antibiotic susceptibility of *Ureaplasma* spp. (Xie and Zhang, 2006; Song et al., 2014). Additionally, 267 *M. hominis* isolates recovered from outpatients between January 2005 and December 2012 were included to determine the trend in the antibiotic susceptibility of *M. hominis* (Kong et al., 2016).

### Sample collection, culture, and antibiotic susceptibility testing

Urethral specimens of male patients were obtained by inserting Dacron swabs 2–3 cm into the urethra and spinning for 5 s, and cervicovaginal specimens of female patients were obtained from the cervical area after exocervical mucus was cleansed with a swab. A commercial Mycoplasma IST2 assay (bioMe’rieux, Marcy-l’E’ toile, France) was used for the identification, semi-quantification of the concentration, and antibiotic susceptibility testing of *Ureaplasma* spp. and *M. hominis*. The specimens were inoculated and incubated according to the manufacturer’s instructions. Briefly, urethral and cervical swabs were inoculated in R1 medium, and the mixture was added to R2 medium and vortexed until the pellet dissolved. Then, the rehydrated R2 growth medium was distributed into wells on the Mycoplasma IST2 strip and protected from drying with mineral oil. The strip and the remaining broth were incubated at 37°C for 48 h and color changes were recorded at 24 h for *Ureaplasma* spp. and 48 h for *M. hominis*. Positive results were noticed when the color of the broth changed from yellow to red with an estimated density of each organism ≥10^4^ CFU.

Antibiotic susceptibility testing was performed for the following antibiotics: ofloxacin, ciprofloxacin, erythromycin, clarithromycin, azithromycin, josamycin, tetracycline, doxycycline, and pristinamycin. The antibiotic resistance breakpoints for the above nine antibiotics (mg/L) were as follows: ofloxacin, resistant (*R*) ≥ 4; ciprofloxacin, *R* ≥ 2; erythromycin, *R* ≥ 4; clarithromycin, *R* ≥ 4; azithromycin, *R* ≥ 4; josamycin, *R* ≥ 8; tetracycline, *R* ≥ 8; doxycycline, *R* ≥ 8; and pristinamycin, *R* ≥ 2 (Kenny and Cartwright, 2001).

### Statistical analysis

The SPSS Statistics for Windows v.21.0 was used to analyze the prevalence and occurrence of resistance to the nine antibiotics tested based on the Chi-square test and Fisher’s exact test. *p-*values of <0.05 were considered significant statistically.

## Results

### Prevalence of *Ureaplasma* spp. and *Mycoplasma hominis* from 2013 to 2019

Among the 135,263 specimens tested, the overall positive rate of total *Ureaplasma* spp./*M. hominis* was 38.1% (51,504 out of 135,263). *Ureaplasma* spp. infection was more common than *Ureaplasma* spp./*M. hominis* coinfection (31.3% vs. 6.0%, *p* < 0.001) and *M. hominis* infection (31.3% vs. 0.8%, *p* < 0.001). Of the 48,638 specimens obtained from male outpatients, 12,266 (25.2%) were positive for *Ureaplasma* spp., 216 (0.4%) for *M. hominis*, and 1970 (4.1%) for both *Ureaplasma* spp. and *M. hominis*. Females had a significantly higher prevalence of *Ureaplasma* spp. and *M. hominis* than males (*p* < 0.001). Of the 86,625 specimens obtained from female outpatients, 30,044 (34.7%) were positive for *Ureaplasma* spp., 886 (1.0%) for *M. hominis*, and 6,122 (7.1%) for both *Ureaplasma* spp. and *M. hominis*.

**Figure 1:**
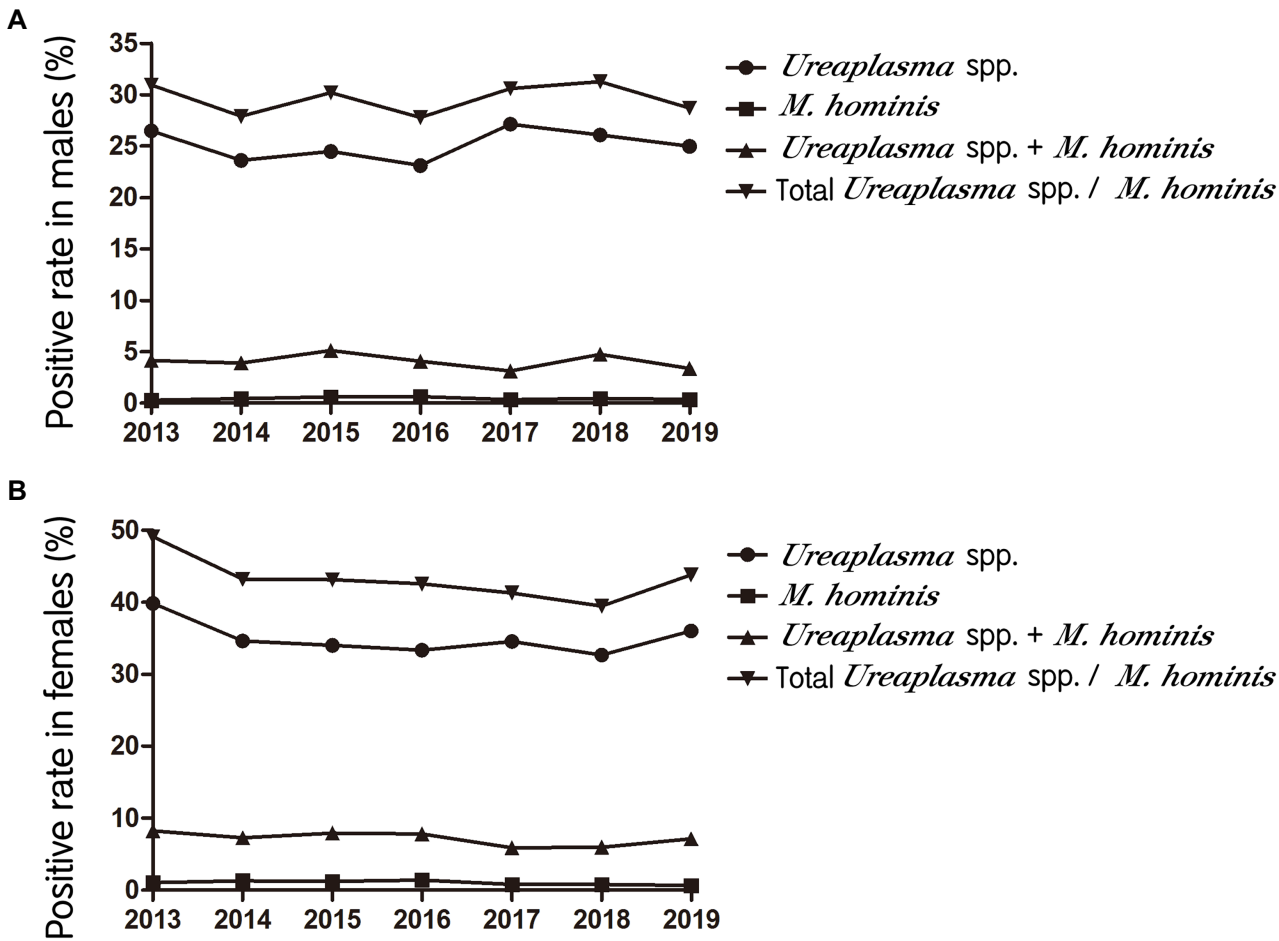
Trends in the prevalence of *Ureaplasma* spp. and *Mycoplasma hominis* in males **(A)** and females **(B)** from 2013 to 2019.

Trends in the prevalence of *Ureaplasma* spp. and *M. hominis* during the test period are shown in Figure 1. *Ureaplasma* spp. infection rates were ranged from 23.1 to 27.1% in males and from 32.7 to 39.9% in females, which were higher than those of *M. hominis* infection and *Ureaplasma* spp./*M. hominis* coinfection (*M. hominis*, 0.3–0.6% for males and 0.8–1.4% for females; coinfection, 3.1–5.1% for males and 5.9–8.2% for females).

### Distribution of *Ureaplasma* spp. and *Mycoplasma hominis* in different age groups from 2013 to 2019

The distribution of *Ureaplasma* spp. and *M. hominis* according to the age group from 2013 to 2019 is presented in Table 1. The overall positive rate of total *Ureaplasma* spp./*M. hominis* was highest in male patients aged 14–20 years (33.6%) and lowest in male patients aged ≥51 years (25.1%), with a declining trend as age increased. Similarly, the overall positive rate of total *Ureaplasma* spp./*M. hominis* was highest in female patients aged 14–20 years (58.0%), followed by 46–50 years (54.7%), and lowest in female patients aged 50–94 years (36.7%). For *Ureaplasma* spp. infection, the highest positive rates were found in both male and female patients aged 14–20 years, with 28.2% for males and 40.2% for females. For *M. hominis* infection, the detection rate was highest in males aged 14–20 years (1.1%) and ≥ 51 years (0.9%), but it occurred most commonly in females aged 46–50 years (2.2%) and ≥ 51 years (2.2%). Notably, among the patients with *Ureaplasma* spp./*M. hominis* coinfection, the highest detection rate was found in females aged 14–20 years (16.8%) and 46–50 years (14.4%); however, close detection rates were found in males of different age groups, ranging from 3.8 to 4.9%.

**Table 1:** Distribution of *Ureaplasma* spp. and *M. hominis* in the two sexes by age from 2013 to 2019.

Specimen	Distribution [no. (%)]			
	*Ureaplasma* spp. (*n* = 42,310)	*M. hominis* (*n* = 1,102)	*Ureaplasma* spp. + *M. hominis* (*n* = 8,092)	Total *Ureaplasma* spp./*M. hominis*
Male (year)				
14–20 (*n* = 372)	105 (28.2)	4 (1.1)	16 (4.3)	125 (33.6)
21–25 (*n* = 2,932)	727 (24.8)	15 (0.5)	111 (3.8)	853 (29.1)
26–30 (*n* = 14,201)	3,645 (25.7)	61 (0.4)	557 (3.9)	4,263 (30.0)
31–35 (*n* = 15,589)	3,961 (25.4)	57 (0.4)	616 (4.0)	4,634 (29.7)
36–40 (*n* = 8,508)	2,149 (25.3)	36 (0.4)	347 (4.1)	2,532 (29.8)
41–45 (*n* = 4,048)	1,014 (25.0)	22 (0.5)	199 (4.9)	1,235 (30.5)
46–50 (*n* = 1,651)	393 (23.8)	9 (0.5)	72 (4.4)	474 (28.7)
≥51 (*n* = 1,337)	272 (20.3)	12 (0.9)	52 (3.9)	336 (25.1)
Total (*n* = 48,638)	12,266 (25.2)	216 (0.4)	1970 (4.1)	14,452 (29.7)
Female (year)				
14–20 (*n* = 572)	230 (40.2)	6 (1.0)	96 (16.8)	332 (58.0)
21–25 (*n* = 9,439)	3,697 (39.2)	111 (1.2)	791 (8.4)	4,599 (48.7)
26–30 (*n* = 31,218)	11,089 (35.5)	258 (0.8)	1915 (6.1)	13,262 (42.5)
31–35 (*n* = 25,467)	8,241 (32.4)	239 (0.9)	1,537 (6.0)	10,017 (39.3)
36–40 (*n* = 11,697)	4,046 (34.6)	131 (1.1)	896 (7.7)	5,073 (43.4)
41–45 (*n* = 4,747)	1717 (36.2)	65 (1.4)	428 (9.0)	2,210 (46.6)
46–50 (*n* = 1,560)	594 (38.1)	34 (2.2)	225 (14.4)	853 (54.7)
≥51 (*n* = 1,925)	430 (22.3)	42 (2.2)	234 (12.2)	706 (36.7)
Total (*n* = 86,625)	30,044 (34.7)	886 (1.0)	6,122 (7.1)	37,052 (42.8)

### Antibiotics effectiveness from 2013 to 2019

The overall resistance rates of *Ureaplasma* spp. and *M. hominis* from 2013 to 2019 are shown in Table 2. Josamycin, tetracycline, doxycycline, and pristinamycin maintained high activity against *Ureaplasma* spp. and *M. hominis*, with resistance rates all <5%. Erythromycin, clarithromycin, and azithromycin were effective against the majority of *Ureaplasma* spp. isolates (resistant rates, <3%). In comparison, ofloxacin and ciprofloxacin displayed limited effectiveness against both *Ureaplasma* spp. and *M. hominis* (resistant rates, >70%).

**Table 2:** Overall resistance rates of *Ureaplasma* spp. and *M. hominis* isolates from 2013 to 2019.

Antimicrobial agents	Resistance rate [*n* (%)]	
	*Ureaplasma* spp. (*n* = 42,310)	*M. hominis* (*n* = 1,102)
Ofloxacin	30,331 (71.7)	923 (83.8)
Ciprofloxacin	37,303 (88.2)	834 (75.7)
Erythromycin	981 (2.3)	/
Clarithromycin	624 (1.5)	/
Azithromycin	554 (1.3)	/
Josamycin	89 (0.2)	19 (1.7)
Tetracycline	629 (1.5)	47 (4.3)
Doxycycline	321 (0.8)	9 (0.8)
Pristinamycin	82 (0.2)	16 (1.5)

### Antibiotic susceptibility patterns of *Ureaplasma* spp. and *Mycoplasma hominis* over 20 years

**Figure 2:**
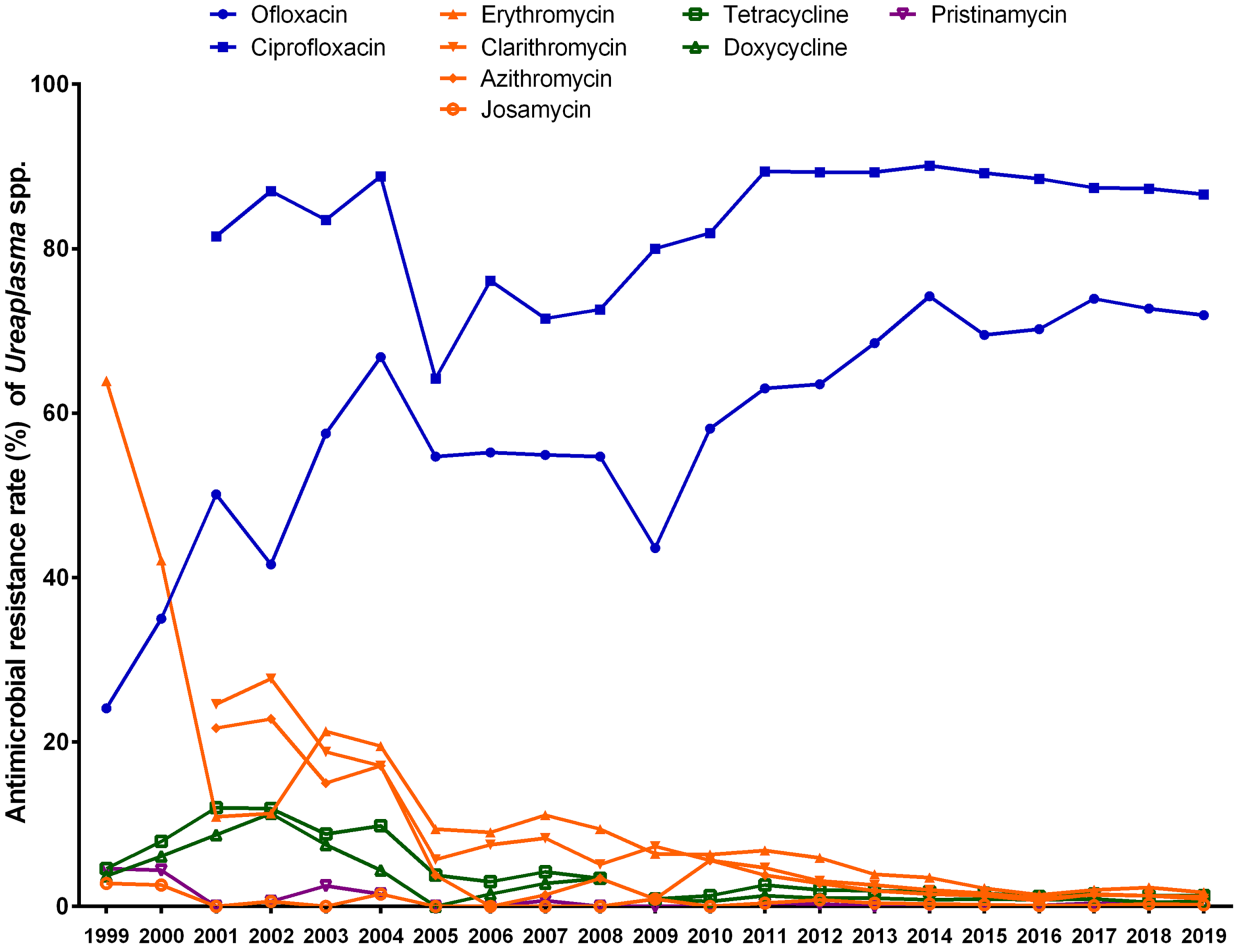
Trends in the antibiotic resistance rates of *Ureaplasma* spp. to nine antibiotics from 1999 to 2019.

The antibiotic susceptibility of *Ureaplasma* spp. isolates collected during 2013–2019 was compared to those collected during 1999–2004 and during 2005–2012 (Figure 2). Ofloxacin resistance of *Ureaplasma* spp. increased from 1999 (resistance rate, 24.1%) to 2019 (resistance rate, 71.9%), whereas ciprofloxacin resistance maintained high from 2001 to 2019, with resistance rates ranging from 64.2 to 93.2% (*p* < 0.001). Resistance to erythromycin, clarithromycin, and azithromycin decreased, with the exception of josamycin, which maintained extremely low (resistance rates, 0–2.8%) during the test period. Resistance to tetracycline and doxycycline increased from 1999 (tetracycline, 4.6%; doxycycline, 3.7%) to 2001 (tetracycline, 12%) or 2002 (doxycycline, 11.3%), then decreased to 2019 (tetracycline, 1.3%, *p* < 0.001; doxycycline, 0.6%, *p* < 0.001). Additionally, resistance rates to pristinamycin were low, ranging from 0 to 4.6%.

**Figure 3:**
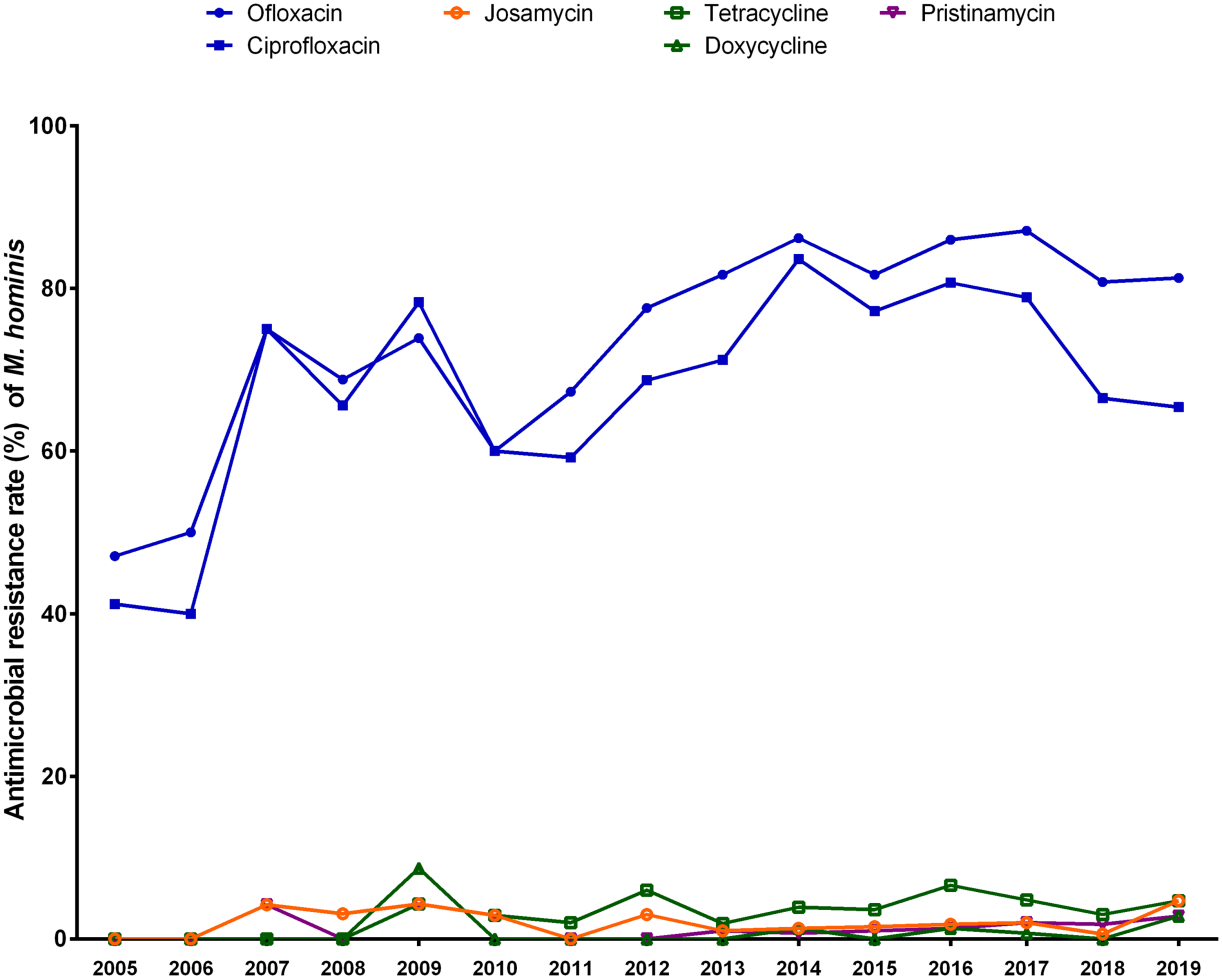
Trends in the antibiotic resistance rates of *Mycoplasma hominis* to six antibiotics from 2005 to 2019.

The trend in the antibiotic susceptibility of *M. hominis* isolates during 2005–2019 is shown in Figure 3. Resistance to ofloxacin and ciprofloxacin rose from 2005 (ofloxacin, 47.1%; ciprofloxacin, 41.2%) to 2019 (ofloxacin, 81.3%; ciprofloxacin, 65.4%), with peaks in 2017 for ofloxacin (87.1%, *p* < 0.001) and 2014 for ciprofloxacin (83.6%; *p* < 0.001). Resistance rates to josamycin, tetracycline, doxycycline, and pristinamycin remained low, ranging from 0 to 8.7%.

## Discussion

*Ureaplasma* species and *M. hominis* are frequent colonizers in the urogenital tract of adults but are sometimes associated with a variety of diseases. This study aimed to evaluate the prevalence and antibiotic susceptibility of these species between 2013 and 2019. Our findings identified a high prevalence of *Ureaplasma* species and *M. hominis* (overall prevalence, 38.1%). *Ureaplasma* spp. infection was the most common (31.3%), followed by *Ureaplasma* spp./*M. hominis* coinfection (6.0%) and single *M. hominis* infection (0.8%).

Compared to our previous study analyzing the period of 2005 to 2013, the positive rates of both *Ureaplasma* spp. and *M. hominis* were decreased in females but increased in males in this study (Song et al., 2014). High prevalence of genital mycoplasmas was also observed in other provinces of China. The positive rates of genital mycoplasmas were detected in 33.9% of female outpatients in Beijing, 38.7% of infertile men in Shanghai, and 47.11% of outpatients for gynecologic healthcare screening or the presence of urogenital infection symptoms in Xi’an (Wang et al., 2016; Zeng et al., 2016; Zhou et al., 2018). Similar results were reported in South Korea, Russia, and Romania (Rumyantseva et al., 2019; Lee and Yang, 2020; Doroftei et al., 2021), but relatively lower positive rates were reported in Poland, Italy, and Brazil (Ponyai et al., 2013; Foschi et al., 2018; Piscopo et al., 2020), which could be explained by the discrepancy in socioeconomic conditions, living standards, and the experimental methods used. Notably, the identification of *Ureaplasma* spp. and *M. hominis* in clinical specimens depends on a variety of commercial Mycoplasma testing kits based on molecular or culture methods, the sensitivity and specificity of which are mostly unknown. Moreover, an unequal prevalence between sexes was observed, in which the detection rates of *Ureaplasma* spp. and *M. hominis* were higher in the female population than in the male population. The higher occurrence of *Ureaplasma* spp. and *M. hominis* in females appears to be a general trend, as evidenced by an increasing number of studies (Ponyai et al., 2013; Zeng et al., 2016; Foschi et al., 2018; Kasprzykowska et al., 2018).

Our study also indicated that the prevalence of *Ureaplasma* spp. was higher in younger individuals and declined with age, but we cannot ignore the fact that the number of both male and female patients aged 14–20 years was considerably lower than that of any other age group. However, *M. hominis* was more frequently isolated from the older individuals, with an increasing trend as age increased, especially in female patients. This result is consistent with our previous study, which showed that *M. hominis* was more prevalent in male patients aged 56–60 years, and in female patients aged 61–65 years and 46–50 years (Kong et al., 2016). Lee et al. reported that *Ureaplasma* spp. were most commonly found in female patients aged 18–29 years, but *M. hominis* was more common in females aged 60–89 years, followed by 30–39 years, in Seoul, South Korea (Lee and Yang, 2020). However, Zhou et al. showed that *Ureaplasma* spp. and *M. hominis* occurred mostly in infertile men aged 26–30 years and 21–25 years, respectively, in Shanghai, China (Zhou et al., 2018). These findings suggest that *Ureaplasma* spp. are more likely to be detected in younger patients, but further studies are required to determine the association between *M. hominis* prevalence and age.

Antibiotic resistance is the leading cause of treatment failure in genital mycoplasmas infections, and the increasing antibiotic resistance has prompted researchers to conduct ongoing monitoring investigations. In this study, a significant variation in levels of sensitivity to various antibiotics was discovered. The majority of clinical *Ureaplasma* spp. isolates were susceptible to macrolides (erythromycin, clarithromycin, azithromycin, and josamycin), tetracyclines (tetracycline and doxycycline), and streptogramins (pristinamycin), suggesting that macrolides, tetracyclines, and streptogramins are effective antibiotics against *Ureaplasma* spp. However, the current findings revealed that *Ureaplasma* spp. were extremely resistant to fluoroquinolones, which is consistent with our and other recent studies on fluoroquinolone resistance in *Ureaplasma* spp. in China (Xie and Zhang, 2006; Song et al., 2014; Wang et al., 2016; Yang et al., 2020; Ma et al., 2021). In our recent study, the resistance rates of levofloxacin were 84.69% for *U. parvum* and 82.43% for *U. urealyticum*, and those of moxifloxacin were 51.44% for *U. parvum* and 62.16% for *U. urealyticum* (Yang et al., 2020). Notably, fluoroquinolone resistance levels differed significantly between countries. In Italy, 77.1% of *Ureaplasma* spp. were ciprofloxacin-resistant, and 26.3% of isolates were ofloxacin-resistant (Foschi et al., 2018). In the United States, however, the resistance rates of levofloxacin in *Ureaplasma* spp. were extremely low, with only 1.6% for *U. parvum* and 0% for *U. urealyticum* (Valentine-King and Brown, 2017). The primary variation is perhaps related to the strategy or inclination for using antibiotics in different regions.

*M. hominis* is intrinsically resistant to C14- and C15-membered macrolides (erythromycin, clarithromycin, and azithromycin), but susceptible to C16-membered macrolides (josamycin). Our results showed that the majority of clinical *M. hominis* isolates were susceptible to C16-membered macrolides (josamycin), tetracyclines (tetracycline and doxycycline), and streptogramins (pristinamycin), but most of them were resistant to fluoroquinolones (ofloxacin and ciprofloxacin). These findings were consistent with several previous studies (Wang et al., 2016; Zeng et al., 2016; Foschi et al., 2018) but differed from others (Valentine-King and Brown, 2017; Foschi et al., 2018). During the test period in China, fluoroquinolone resistance increased and reached an extraordinarily high level against both *Ureaplasma* spp. and *M. hominis*, perhaps due to the inappropriate use of fluoroquinolone agents in both poultry industry and clinical settings (Chen Z. et al., 2021; Chen H. et al., 2021).

Overall, our results showed that josamycin, tetracycline, doxycycline, and pristinamycin maintained outstanding activity against *Ureaplasma* spp. and *M. hominis*. Due to its toxicity, pristinamycin was no longer a viable alternative, so it has been unavailable for therapeutic prescription in several countries. Additionally, erythromycin, clarithromycin, and azithromycin are all candidates for *Ureaplasma* spp. infection therapy.

This study has some important limitations. First, it was unable to discriminate between actual genital mycoplasma infection and common commensal colonization due to the lack of clinical data on the participants. Second, the Mycoplasma IST2 kit failed to separate between *Ureaplasma* spp. (*U. parvum* and *U. urealyticum*), as well as produce distinct findings for mixed cultures of *Ureaplasma* spp. and *M. hominis*, which might result in inaccurate reporting of antibiotic resistance. Third, since all clinical isolates of *Ureaplasma* spp. and *M. hominis* were generated as part of routine clinical laboratory procedures and were disposed of after being tested, the identification and antibiotic susceptibility results produced by the Mycoplasma IST2 kit cannot be compared with some other molecular-based methods or the standardized guidelines of the Clinical and Laboratory Standards Institute (CLSI) on Antimicrobial Susceptibility Testing. Regrettably, the antibiotics and breakpoints used in the Mycoplasma IST2 kit conflict with CLSI recommendations. Fourth, we were unable to perform further studies to determine the mechanisms of resistance to fluoroquinolones, macrolides, and tetracyclines in *Ureaplasma* spp. and *M. hominis*.

In conclusion, our study retrospectively analyzed the prevalence and antibiotic susceptibility of *Ureaplasma* spp. and *M. hominis* in Hangzhou, China, from 2013 to 2019. *Ureaplasma* spp. infection was relatively common, but *M. hominis* infection and *Ureaplasma* spp./*M. hominis* coinfection were exceedingly rare. Furthermore, both *Ureaplasma* spp. and *M. hominis* were more prevalent in females than in males, and their distribution was associated with age. Josamycin, doxycycline, and tetracycline are promising antibiotics with outstanding activity against *Ureaplasma* spp. and *M. hominis*.

## Data availability statement

The original contributions presented in the study are included in the article/supplementary material, further inquiries can be directed to the corresponding authors.

## Ethics statement

This study was approved by the local Research Ethics Committee of Sir Run Run Shaw Hospital, Zhejiang University School of Medicine, China. All isolates were generated as part of routine clinical laboratory procedures, and no identifiable patient information was collected.

## Author contributions

JS, XX, and JZ designed experiments. JS and XW carried out experiments and analyzed the results. YK and HJ checked data. JS, TY, XX, and JZ wrote the manuscript. All authors contributed to the article and approved the submitted version.

## Funding

This study was supported by the National Natural Science Foundation of China (82072342 and 82102429).

## Conflict of interest

The authors declare that the research was conducted in the absence of any commercial or financial relationships that could be construed as a potential conflict of interest.

## Publisher’s note

All claims expressed in this article are solely those of the authors and do not necessarily represent those of their affiliated organizations, or those of the publisher, the editors and the reviewers. Any product that may be evaluated in this article, or claim that may be made by its manufacturer, is not guaranteed or endorsed by the publisher.
